# Demand-side determinants of timely vaccination of oral polio vaccine in social mobilization network areas of CORE Group polio project in Uttar Pradesh, India

**DOI:** 10.1186/s12879-018-3129-2

**Published:** 2018-05-16

**Authors:** Manojkumar Choudhary, Roma Solomon, Jitendra Awale, Rina Dey

**Affiliations:** CORE Group Polio Project – India, 303, Bestech Chambers, B-Block, Sushant Lok-I, Gurgaon, Haryana 122002 India

**Keywords:** Polio, Timely immunization, CGPP, India

## Abstract

**Background:**

Children who receive all doses of scheduled vaccines reduce their susceptibility to vaccine-preventable diseases. In India, full immunization coverage has increased significantly. However, only a small proportion of children are immunized on time. Globally, studies on factors affecting coverage of childhood immunization have found a significant impact by demand and supply-side determinants. This paper explores the demand-side determinants of timely immunization of the third dose of oral polio vaccine (OPV3) among children aged 6–11 months in the catchment areas of CORE Group Polio Project India.

**Methods:**

We analyzed secondary de-identified data from a household level ‘Doers and Non-doers survey’ conducted in 2015. Determinants of timely OPV3 immunization were identified by modeling the characteristics of index children and survey respondents, surveyed households, respondents’ media habits, their exposure to immunization services and perceptions towards child immunization, through a multinomial regression analysis.

**Results:**

The eight demand-side predictors based on the background characteristics and perceptions of caregivers determined timely vaccination of OPV3. The strongest predictor of timely OPV3 immunization was found to be the fathers’ educational level. Children of uneducated or lesser educated fathers had increased odds of not receiving the OPV1 vaccination, as compared to children of more educated fathers (OR > 10). Respondents who strongly perceived other (non-health) benefits of child immunization were three times more likely to timely vaccinate their children than those who do not. Furthermore, mothers who disagreed with the positive attributes of child immunization were 25 times more likely to delay or not to take their children for OPV immunization on time.

**Conclusions:**

This study found eight essential factors that are responsible for timely OPV3. Despite limitations in data collection and analysis, immunization programs in India could use the eight identified demand-side determinants of timeliness and tailor communication strategies accordingly. We suggest that program communication efforts be directed at male community members; such messaging should address parents’ perceptions of non-health benefits and stress the positive attributes of child immunization. Further investigation would be helpful to assess the various risk factors of under-vaccination as well as vaccinators’ understating about timely immunization.

**Electronic supplementary material:**

The online version of this article (10.1186/s12879-018-3129-2) contains supplementary material, which is available to authorized users.

## Background

Worldwide, tremendous progress has been reported in the reduction in the numbers of child deaths. Routine childhood immunization has proven to be among the most practical and most cost-effective health interventions [1–3]. Vaccination has contributed to saving millions of lives each year, resulted in the eradication of smallpox, reduced the global incidence of polio by 99% and lowered morbidity and mortality from diphtheria, tetanus, whooping cough, measles, Haemophilus influenza type B and meningococcal A meningitis [3]. In India, an increase has been observed in the overall coverage of childhood routine immunization (RI); at the national level, full immunization coverage has increased from 44% in 2005–06 to 62% in 2015–16 [4].

Timeliness is an essential component of child immunization that substantially reduces susceptibility to vaccine-preventable diseases (VPDs). An adequate number of on-time immunized children is the primary requirement for interrupting disease transmission. Delayed vaccination against childhood diseases, therefore, is known to result in increased rates of mortality and morbidity [5]. A study based on district level household survey (DLHS–3) data reported that a lack of timeliness vaccination of key childhood vaccines like DPT3 and MCV1 is one of the main challenges in India. This study found that only a small proportion of children aged 0–60 months in India are immunized on time: 30% of children received BCG vaccine by 4 weeks (recommended at birth); 28% received DPT1 by 8 weeks (recommended at 6 weeks); and only 12% received MCV1 by the recommended age of 9 months [6]. Another study conducted in high immunization coverage areas of South India found that delay in vaccination for more than two weeks from the due date for DPT1, DPT2, DPT3 and measles was 7.4, 41.9%, 6 4.4 and 38.8%, respectively [7].

Project administrative data and periodic external evaluations of CORE Group Polio Project (CGPP)^1^ from Uttar Pradesh, a northern state of India, showed a significant increase in RI coverage. The percentage of fully immunized children (received BCG, 3 DPTs, 3 OPVs and Measles) in the catchment areas of CGPP India has increased from 64% in 2010 to 78% in 2015 [8, 9]. In 2014, an analysis of administrative data of CGPP India found that only 19% of children received OPV3 at the ages of 14–20 weeks. However, OPV3 coverage among children aged 12–23 months was 86% [10, 11]. This identified gap between the crude and timely immunization with OPV3 led to the query ‘Why are children not getting immunized on time?’

Worldwide, individual studies and systematic reviews found a myriad of factors determines the coverage of child immunization [1, 5, 12–25]. One systematic review of low and middle-income countries categorized the determinants of vaccination coverage into three groups: intent to vaccinate, health facility readiness, and community access [26]. Known determinants of child immunization can be broadly categorized into two categories: ‘Supply-side’ and ‘Demand-side’.

In India, childhood immunization coverage differs by states and districts [27–29]. Districts’ per-capita income is a strong predictor of better vaccination outcomes for children [27]. A review of studies conducted across India found that availability and access to health/immunization services [7, 29, 30], attitudes and practices of health/immunization staff [30, 31], quality/reliability of health/immunization services [30] and supply of vaccines and logistics [31] are the main supply-side factors affecting vaccination coverage. Identified demand-side determinants are: characteristics of children (gender [29, 32, 33] and birth order [7, 29, 34]); maternal characteristics (age [34], education level [27, 28, 32, 33, 35, 36], occupation/employment status [34, 37]), place of delivery [31, 32, 38], ante-natal visits [32], mothers’ tetanus immunization status during the previous delivery [28, 31, 32], exposure to communication interventions [14]); parents’ characteristics (education level [28, 29, 31, 38]); parents’, knowledge of immunization schedule [30, 33, 36, 37, 39], their attitudes/perceptions towards immunization [30, 37, 40], fear of side effects [30, 36], conflicting priorities and beliefs [30]); characteristic of families (family type/family size [31], religion [29, 32, 38], caste [29, 32], socio-economic status of households/household wealth index [28, 29, 33]); and community level factors (place/area of residence [29, 32], community norms/customs and location/distance to immunization site/health care services [28]). In a nutshell, supply-side factors largely affect the reach of vaccination services and influence the vaccination status of children (vaccinated or not vaccinated). Drop-out in vaccination of vaccines that are given in a series (e.g. DPT, Penta, OPV, Measles) is mostly affected by demand-side factors rather than by supply-side factors [35].

An information gap and an apprehension of an adverse event following immunization (AEFI) were found to be the two main reasons (22 and 23%, respectively) for children not being fully immunized (i.e., unvaccinated or dropped-out children) [41], according to RI monitoring data^2^ from Uttar Pradesh in May 2014. Vaccine acceptance can be undermined by ignorance or negative attitudes of caregivers towards vaccination by indicating inadequate and inappropriate information sharing [42] and poor communication [43]. An effective communication response to address the awareness gap requires a better understanding of the target audience and key messages [44–47]. However, a review of literature found insufficient information about appropriate target audience and content of messages on program communication for increasing timeliness of childhood immunization, particularly among Indian children. Responding to this information gap, along with other information needs of the project, CGPP India conducted an in-depth investigation for effectively designing its communication interventions and revamping the strategies to generate demand for routine polio immunization. Although DPT is the commonly used vaccine for assessing coverage or determinants of RI, this particular study used OPV instead. Along with RI, children under five years old in India were also vaccinated through polio vaccination campaigns (supplementary immunization activities - SIAs). OPV vaccination through RI becomes very crucial in building/maintaining population immunity against polio, as the frequency of polio SIAs is minimized to four-five activities per year.

According to the latest national immunization schedule of India, children should be vaccinated with polio vaccine at birth (within two weeks of birth, as OPV0 dose), 6 weeks (OPV1 & IPV1), 10 weeks (OPV2), 14 weeks (OPV3 & IPV2) and 16–24 months as OPV booster. Most of the previously conducted studies assessed determinants of vaccination coverage either by comparing ‘Vaccinated children vs. Unvaccinated’ or ‘Dropped-out vs. No drop-out’ [7, 14, 23–25, 27–29, 31, 32, 34, 35, 37, 39]. Our earlier analysis has also explored the determinants of routine immunization performance by analyzing drop-out rate of DPT3 in CGPP India areas [14]. However, the CGPP India functionaries observed that the profile of ‘Not at all vaccinated children’ differs from ‘Dropped-out children’. In this paper, we examine the influence of caregivers’ characteristics and perceptions in timeliness of OPV immunization by comparing ‘timely vaccinated children’ with ‘unimmunized and dropped-out children’. Our main hypothesis is that demand-side determinants of timely OPV3 immunization are likely to be different from determinants of routine immunization performance (measured in the form of ‘Vaccinated vs. ‘Unvaccinated’).

## Methods

### Study design

This paper is based on secondary analysis of de-identified data, originally collected on the principles of ‘Doers and Non-doers survey’^3^. The study followed a case-control type design.

### Study area and description of data

Data was collected from the catchment areas of CGPP India, consisting of 44 work areas of community mobilization coordinators^4^ (villages/urban areas) from three districts of Uttar Pradesh. The observed level of full immunization coverage (FIC)^5^ among children aged 12–23 months (through RI monitoring) was the key criterion used for the selection of study districts. The three selected study districts represented the high (Saharanpur district, FIC was 68%), average (Rampur district, FIC was 54%) and low (Sambhal district, FIC was 41%) levels of FIC from among the 12 catchment districts of CGPP India [41].

#### Defining doers and non-doers of the study

Time-appropriate vaccination is measured either at the exact recommended age of a particular vaccine or by adding additional time after the scheduled immunization date for an individual vaccine [7, 48]. This study collected information for children age 6–11 months to collect sufficient numbers of samples among the unvaccinated, dropped-out and timely vaccinated children. ‘OPV vaccination status of children aged 6-11 months was the key indicator to determine the study respondents. The ‘Doers’ were defined as mothers of timely immunized children (children aged 6–11 months who received the third dose of OPV vaccine before reaching the age of four months). The ‘Non-doers’ were divided into two categories: mothers of children who have not received the first dose of OPV and mothers of children who received only the first or second dose of OPV (representing actual/potential dropout). These three non-overlapping subgroups of children were considered as strata.

#### Recruitment of study respondents

Samples were selected in two stages from each of the three independent strata. In the first stage, 10 clusters (CMC work areas) were randomly selected from each of the study districts. As data collectors did not locate the desired number of children without OPV1 immunization (non-doers), more clusters were added to result in 44 clusters. From every selected cluster (CMC area), investigators listed children aged 6–11 months and recorded each child’s vaccination history. The survey respondents were exclusively selected on the basis of recorded OPV vaccination status in the immunization (RI) cards^6^ to avoid possible misclassification bias (respondents’ limitations in the recall of vaccination dates/age at vaccination) in the selection of ‘Doers’ and ‘Non-doers’. Children who did not have any type of RI cards were excluded from the study.

A total of 611 children were randomly selected from three sampling frames, comprising 266 children who received timely immunization of OPV3 (Doers), 72 children without OPV1 immunization (Non-doers – type A) and 273 who received OPV1 or OPV2 (Non-doers - type B). Mothers of these children were contacted and requested to participate in the study. Out of 611 prospective respondents, a total of 583 face-to-face interviews of mothers were conducted, consisting of 254 doers, 68 non-doers–type A and 261 non-doers- type B. The survey included interviews with 583 mothers representing about 50,000 mothers of children aged 6–11 months from the catchment areas of CGPP India (See Additional file 1: Table S1).

Trained investigators collected the data from May 19 to June 27, 2015. Data was collected through a pre-tested semi-structured interview instrument. The survey data included information about the following: immunization status of index children^7^; mothers’ awareness about RI status of index children; mothers’ perceptions of positive/negative consequences of child immunization, social norms about immunization, child’s susceptibility and severity of selected VPDs, delivery of RI services; mothers’ exposure to RI services; her information sources on child immunization and media habits, lifestyle related information; background characteristics of survey households (house type, household size, family type, ownership of domestic goods/facilities), respondent mothers (age, education, marital status, working status, religion), respondents’ spouse (age, education and working), chief wage earner in the household (age, education, occupation) and index children (age, sex and place of birth).

### Data processing and statistical analysis

The survey dataset was analyzed using the statistical software, IBM SPSS, version 20. A descriptive overview of survey data is presented using frequency distribution and cross-tabulations. OPV immunization status of children aged 6–11 months is the **outcome** (dependent) **variable** of the study, which is polytomous with three categories, i.e., a) not received the first dose of OPV, b) received first or second dose of OPV and c) received three doses of OPV on time.

As the study samples were selected using a multi-stage sampling, a complex samples analysis option available in IBM SPSS 20 was used to identify determinants of OPV immunization outcomes (No OPV1, OPV1/OPV2, and OPV3) by performing exploratory analysis and multinomial logistic regression [49]. Using a ‘Complex Sample Analysis’ tool, we first used univariate logistic regression to identify determinants (our covariates) associated with the outcome variable. Odds Ratios (OR) and the corresponding 95% confidence intervals (CI) estimated the strength of association. Potential determinants included socio-demographic variables, media habits, lifestyle-related practices, exposure to immunization activities/services, interactions with health care functionaries, exposure to specific Information, Education and Communication (IEC) materials^8^ of CGPP and perceptions on child immunization and health.

All the explanatory variables (covariates) found significantly associated (*p*-value < 0.05) with the dependent variables in univariate logistic regression analysis were considered for multinomial regression analysis. All the covariates were assessed for multicollinearity before including in the multinomial regression model. The religion and gender of index children are the known risk factors for immunization coverage, but the univariate logistic regression analysis did not find a significant association of these factors with the outcome variable. Hence, the final multinomial model included all the significantly associated factors of univariate analysis, and it was adjusted for the two confounders of religion and gender of index children. While performing multinomial logistic regression analysis, all the predictors were transformed into categorical variables and results were given for no OPV1 and OPV1/OPV2 immunization with reference to timely OPV3 immunization. The performance of Model was estimated with the Nagelkerke Pseudo-*R*2 statistic (a measure of explained variation in the model). Cases with missing data were excluded from all the analysis.

## Results

Background characteristics of three independent samples of doers and non-doers survey are presented in Additional file 1: Table S2 to Table S6. Out of 583 surveyed index children, 54% were male and 46% female. About 56% of children were born in institutions (government or private hospital). The mean age of the interviewed mothers was 26.2 years. The majority of mothers (62%) reported having no formal education. Almost all (99%) mothers stayed at home, and a large proportion (94%) of respondents reported doing no income generation work. The majority (83%) of the interviewed mothers were Muslims. The mean age of the husbands of respondents was 30.0 years. About half of the respondents’ husbands had formal education, and 21% of them had completed schooling of grade (standard) 10 and above. In contrary to study respondents, almost all (99%) of respondents’ husbands were doing income generation work, and most (91%) of them worked outside of the home. Half of the mothers stayed with joint families, and about two-thirds (64%) of the interviewed mothers stayed in *Pucca* houses (houses made with high-quality materials). A greater proportion of timely immunized children were from households with higher wealth quintile, whereas the children without OPV1 or with OPV1/OPV2 immunization belong to households with comparatively lower wealth quintile.

Regarding media habits of respondents, about one-third of mothers did not have exposure to print or electronic media. Television is the most common media, watched by over two-thirds of respondent mothers across the three sample groups. The newspaper is the second common media, read by around 15% of respondents and newspaper readership differs by the independent samples. Less than one-tenth of respondents reported listening to the radio, and a negligible proportion of interviewed mothers traveled to cinema halls to watch movies. The survey respondents had limited mobility from their place of residence. About two-thirds of interviewed mothers have mobile phones in their households. Less than half of the mothers can use it on their own, and a small proportion of them could even send text messages.

Over three-fourths of the mothers reported ever visiting the sites of RI or supplementary immunization activities (SIAs). The proportion of mothers who ever visited any immunization site is considerably lower among the mothers of not immunized children, compared to timely immunized children. A large proportion of interviewed mothers stated that they ever discussed the importance of childhood immunization with the local health provider, including CMCs deployed by CGPP India (See Additional file 1: Table S7).

The majority (> 80%) of mothers from all the three independent samples stated that the ‘CMC’ was an information source for childhood immunization. Compared to the mothers of unimmunized children, more mothers of timely immunized children stated that they learned about the upcoming vaccination (due immunization) through the local health care providers (other than CMCs) as well as from their family members or neighbors. More than two-thirds of respondents said they had been exposed to at least one CGPP Indian-developed IEC resource, during a visit to the immunization session site or routine communication activities of CMCs in the community. More mothers of timely immunized children had more exposure to CGPP India’s IEC material than mothers of unimmunized had children (Refer to Additional file 1: Table S8 and S9). The majority (> 85%) of interviewed mothers heard of VPDs like tuberculosis, polio, tetanus, pertussis, jaundice, diarrhea and measles, but about half had not heard of diphtheria (See Additional file 1: Table S10).

Mothers’ perceptions on the importance of childhood immunization presented in Table 1 significantly vary among the three groups of respondents (Computation details are presented in the Additional file 2). More mothers of timely immunized children perceived benefits of child immunization when compared with the other groups of respondents. The survey respondents disagreed with a statement *‘There is no particular age, week or day; we can get the child immunized at any time’. However,* more mothers of children with timely immunization agree with the positive attributes of child immunization and hospital delivery (See Table 2).

**Table 1:** Perception of respondents on the importance of child immunization by three independent samples of the survey

Perception	Score (quintile)	Percentage^♦^ of mothers of children with:			Overall *(n = 583)*	*p* value^$^
		No OPV1 immunization *(n = 68)*	OPV1/OPV2 immunization *(n = 261)*	Timely OPV3 immunization *(n = 254)*		
Perceived core benefits of immunization	Lower quintile	48.8	32.2	28.5	33.2	0.030*
	Middle quintile	29.2	26.9	41.7	32.6	
	Upper quintile	22.0	40.9	29.8	34.2	
Perceived no harm in immunization and have trust in government initiatives	Lower quintile	28.2	37.3	34.9	35.2	0.200
	Middle quintile	42.0	35.9	29.0	34.2	
	Upper quintile	29.8	26.8	36.1	30.6	
Perceived other (non-heath) benefits of immunization	Lower quintile	55.4	34.7	29.4	35.6	0.045*
	Middle quintile	19.6	33.0	33.7	31.4	
	Upper quintile	25.0	32.3	36.9	33.0	
Perceived that educated/ aware parents go for immunization	Lower quintile	29.2	24.6	44.1	32.3	0.041*
	Middle quintile	26.5	41.5	29.1	34.9	
	Upper quintile	44.3	33.9	26.9	32.8	

^♦^Percentages are weighted by population size, adjusted for stratification, and clustering

^$^*p* value based on chi-square test; *statistically significant at *p* < 0.05; ** *p* < 0.01; ***p < 0.001

**Table 2:** Agreement of respondents on attributes of child immunization and place of delivery by three independent samples of the survey

Perception	Agreement level	Percentage^♦^ of mothers of children with:			Overall *(n = 583)*	*p* value^$^
		No OPV1 immunization *(n = 68)*	OPV1/OPV2 immunization *(n = 261)*	Timely OPV3 immunization *(n = 254)*		
Positive attributes/benefits of child immunization and it is essentials these days (in polluted environment/ contaminated eatables & water)	Strongly disagree/ Disagree	46.9	30.7	10.1	25.4	0.030*
	Neither agree nor disagree	34.2	41.7	38.1	39.4	
	Strongly agree/ Agree	18.9	27.6	51.8	35.2	
Negative attributes/ no benefit of child immunization	Strongly disagree/ Disagree	76.7	86.1	81.4	83.1	0.379
	Neither agree nor disagree	17.3	10.8	15.0	13.2	
	Strongly agree/ Agree	6.0	3.1	3.6	3.7	
Positive attributes of hospital delivery and child immunization is discussed among friends	Strongly disagree/ Disagree	30.1	11.1	11.8	14.0	0.134
	Neither agree nor disagree	30.5	29.7	28.9	29.5	
	Strongly agree/ Agree	39.4	59.2	59.3	56.5	
Child immunization is meant for educated/moving/progressive families	Strongly disagree/ Disagree	27.4	27.3	33.7	29.7	0.446
	Neither agree nor disagree	43.8	30.7	30.3	32.4	
	Strongly agree/ Agree	28.8	41.9	36.0	37.9	
Gender selective immunization	Strongly disagree/ Disagree	89.8	92.9	93.8	92.8	0.016*
	Neither agree nor disagree	6.9	5.9	1.6	4.5	
	Strongly agree/ Agree	3.4	1.2	4.6	2.7	

^♦^Percentages are weighted by population size, adjusted for stratification, and clustering

^$^*p* value based on chi-square test; *statistically significant at *p* < 0.05; ** *p* < 0.01; ***p < 0.001

Responding to the specific question that was enquired only to the non-doer mothers, ‘child was sick’ is the main reason stated by 42% of respondents. About one-fifth (21%) of mothers stated that the child was out of home/village. About 15% of respondents perceived adverse effects of vaccination, and about 8 % of respondents were unaware of the significance. Very minimal proportion (< 2%) of mothers stated the reasons related to the availability and quality of vaccination services (See Additional file 1: Table S11).

### Determinants of OPV immunization

Results of univariable logistics regression analysis found that timely OPV3 immunization is significantly associated with multiple factors like background characteristics of mothers and fathers, wealth index of households and perception of respondents. Table 3 shows the results of the multinomial logistic regression analysis of determinants of timely OPV3 immunization. The main effects model explaining 48% of the variance (Nagelkerke R^2^ = 0.481) identified eight predictors of timely immunization: income generation status of mothers, education of fathers, wealth status of households, newspaper reading habit of mothers, having a mobile phone in the household, mothers’ visit to immunization site, mothers’ agreement with benefits of child immunization and perception that ‘child immunization is essential these days’.

**Table 3:** Predictors of timely OPV3 immunization after multinomial logistic regression

Factors	No OPV1 vs. OPV3®		OPV1/OPV2 vs. OPV3®	
	Unadjusted OR (95% CI)	Adjusted^$^ OR (95% CI)	Unadjusted OR (95% CI)	Adjusted^$^ OR (95% CI)
Maternal age (in years)^[a]^				
< 20 years	6.80 (1.46, 31.72)*	2.23 (0.17, 28.79)	1.37 (0.32, 5.85)	1.12 (0.29, 4.38)
20–24 years	1.10 (0.37, 3.24)	0.45 (0.10, 2.09)	0.82 (0.43, 1.58)	0.76 (0.34, 1.70)
25–29 years	1.26 (0.41, 3.90)	0.58 (0.15, 2.20)	0.97 (0.44, 2.15)	1.04 (0.43, 2.49)
30–34 years	2.62 (0.98, 7.00)	1.04 (0.26, 4.09)	1.03 (0.56, 1.87)	0.83 (0.41, 1.70)
> 34 years	1	1	1	1
Education level of mothers (respondents)				
No formal education	3.83 (1.39, 10.59)*	0.51 (0.13, 1.94)	3.04 (1.37, 6.76)*	1.93 (0.78, 4.77)
Grade (Standard) 1–4	4.93 (1.15, 21.25)*	0.88 (0.15, 4.98)	2.26 (0.49, 10.33)	1.89 (0.44, 8.21)
Grade (Standard) 5–9	1.68 (0.68, 4.19)	0.71 (0.15, 3.32)	1.61 (0.62, 4.19)	2.00 (0.64, 4.24)
Grade (Standard) 10 and above	1	1	1	1
Place of income generation of mothers				
Income generation work from home	0.19 (0.06, 0.56)*	0.12 (0.03, 0.57)*	0.33 (0.16, 0.68)**	0.35 (0.12, 0.99)*
Income generation work from outside home	1.00 (0.29, 3.45)	1.72 (0.31, 9.62)	0.83 (0.32, 2.16)	1.77 (0.47, 6.63)
No income generation work (stay at home)	1	1	1	1
Education level of fathers				
No formal education	21.04 (5.77, 76.73)**	39.63 (7.26, 216.19)**	2.02 (1.09, 3.76)*	0.80 (0.41, 1.59)
Grade (Standard) 1–4	7.05 (2.30, 21.62)**	10.04 (2.04, 49.35)*	0.83 (0.46, 1.50)	0.31 (0.16, 0.60)**
Grade (Standard) 5–9	9.50 (2.36, 38.21)**	17.40 (2.48, 122.03)*	1.13 (0.50, 2.57)	0.65 (0.36, 1.17)
Grade (Standard) 10 and above	1	1	1	1
Wealth quintile of households				
Lowest	4.65 (1.33, 16.28)*	5.07 (0.89, 28.83)	3.40 (1.13, 10.28)*	1.96 (0.49, 7.80)
Second	5.30 (1.89, 14.89)**	3.86 (0.78, 19.00)	2.90 (1.10, 7.66)*	1.56 (0.43, 5.66)
Middle	2.94 (1.15, 7.48)*	2.52 (0.56, 11.29)	1.34 (0.59, 3.03)	1.06 (0.35, 3.22)
Fourth	4.27 (1.53, 11.94)*	4.69 (1.35, 16.30)*	2.25 (0.82, 6.22)	2.33 (0.83, 6.57)
Highest	1	1	1	1
Respondents habit of reading news paper				
Do not read	1.57 (0.47, 5.22)	0.30 (0.12, 0.75)*	3.55 (1.50, 8.45)*	2.75 (1.36, 5.58)*
Read	1	1	1	1
Respondents having a family member, who works in a big metro town (like Delhi)				
No	0.46 (0.23, 0.94)*	0.27 (0.06, 1.18)	1.30 (0.61, 2.81)	1.00 (0.38, 2.65)
Yes	1	1	1	1
Respondents having a mobile phone in the household				
No	1.88 (1.33, 2.66)**	3.35 (1.36, 8.24)*	1.97 (1.35, 2.87)**	1.71 (0.86, 3.41)
Yes	1	1	1	1
Respondents know how to use a mobile phone, use it independently				
No	2.49 (1.36, 4.59)*	0.67 (0.31, 1.44)	2.13 (1.18, 3.85)*	1.06 (0.72, 1.58)
Yes	1	1	1	1
Respondents know how to use mobile for sending messages (SMS)				
No	3.27 (1.03, 10.37)*	1.43 (0.24, 8.36)	4.94 (1.80, 13.51)**	1.65 (0.61, 4.45)
Yes	1	1	1	1
Respondents visited any immunization site (either RI site or polio booth)				
Never visited	8.57 (2.95, 24.87)**	11.30 (3.72, 34.30)**	1.65 (0.69, 3.94)	2.13 (0.90, 5.06)
Ever visited	1	1	1	1
Respondents’ discussion with local health care providers on the importance of child immunization				
Never discussed	5.08 (2.40, 10.76)**	1.83 (0.45, 7.49)	1.87 (1.00, 3.48)	2.19 (0.82, 5.83)
Ever discussed	1	1	1	1
Respondents’ information sources on child immunization (getting a child immunized): Community Mobilization Coordinators (CMC)				
No	2.42 (1.16, 5.04)*	4.45 (0.90, 21.98)	1.83 (0.92, 3.64)	3.40 (0.83, 13.99)
Yes	1	1	1	1
Respondents’ exposure to CGPP India’s IEC materials: CMC Potli				
No	2.79 (1.35, 5.75)*	1.59 (0.39, 6.46)	2.11 (1.05, 4.25)*	1.36 (0.45, 4.13)
Yes	1	1	1	1
Respondents’ exposure to CGPP India’s IEC materials: Flash card				
No	6.04 (2.83, 12.91)**	2.35 (0.78, 7.02)	2.64 (1.36, 5.12)*	1.81 (0.74, 4.43)
Yes	1	1	1	1
Respondents’ exposure to CGPP India’s IEC materials: Flipbook (Aao Jane)				
No	3.48 (1.99, 6.08)**	1.28 (0.43, 3.79)	2.11 (1.24, 3.61)*	1.00 (0.53, 1.86)
Yes	1	1	1	1
Respondents perceived core benefits of immunization				
Lower quintile	2.32 (0.74, 7.27)	1.68 (0.58, 4.85)	0.83 (0.30, 2.24)	0.70 (0.29, 1.69)
Middle quintile	0.95 (0.48, 1.88)	1.29 (0.46, 3.63)	0.47 (0.26, 0.85)*	0.59 (0.30, 1.15)
Upper quintile	1	1	1	1
Respondents perceived no harm in immunization and have trust in government initiatives				
Lower quintile	0.98 (0.44, 2.20)	0.36 (0.07, 1.83)	1.44 (0.60, 3.48)	0.59 (0.13, 2.59)
Middle quintile	1.76 (1.08, 2.88)*	0.31 (1.03)	1.67 (1.15, 2.42)*	0.93 (0.57, 1.53)
Upper quintile	1	1	1	1
Respondents perceived other (non-health) benefits of immunization				
Lower quintile	2.78 (1.74, 4.42)**	3.00 (1.27, 7.12)*	1.35 (0.89, 2.04)	1.53 (0.80, 2.95)
Middle quintile	0.85 (0.34, 2.17)	0.99 (0.38, 2.59)	1.12 (0.51, 2.46)	1.06 (0.61, 1.86)
Upper quintile	1	1	1	1
Respondents’ agreement level with - Positive attributes/benefits of child immunization and it is essentials these days				
Strongly disagree/ Disagree	12.66 (1.46, 109.93)*	25.68 (1.10, 599.24)*	5.70 (0.71, 45.80)	9.17 (0.49, 171.82)
Neither agree nor disagree	2.46 (0.84, 7.19)	2.55 (0.66, 9.80)	2.06 (0.74, 5.76)	2.17 (0.71, 10.34)
Strongly agree/ Agree	1	1	1	1
Respondents’ agreement level with – Gender selective immunization				
Strongly disagree/ Disagree	1.31 (0.32, 5.38)	3.94 (0.26, 59.61)	3.78 (1.23, 11.64)*	2.24 (0.61, 8.19)
Neither agree nor disagree	5.75 (0.46, 72.12)	8.34 (0.46, 152.97)	13.71 (1.57, 119.63)*	6.38 (0.58, 70.00)
Strongly agree/ Agree	1	1	1	1

*n* = 575; Model explaining 48% of variance (Nagelkerke = 0.481)

**®**Reference category: OPV3 (timely immunization of OPV3); * statistically significant at *p* < 0.05; ** *p* < 0.01; ***p < 0.001; Missing data: [a] = 1 (0.2%)

^$^Adjusted for gender of index children, religion of mothers and other covariates of model

Fathers’ education is the strongest predictor of timely OPV immunization. Children of uneducated or less educated fathers had increased odds of ‘No OPV1 immunization’, as compared to the children of more educated fathers. Children of mothers who never visited any immunization site are 11 times more unlikely to immunize in a timely manner for OPV3. Children of mothers who are engaged in income generation activities are more likely to be immunized on time, compared to the children of mothers without any income-generation work. Children from the lower wealth quintile are more likely not to receive OPV1. Children from households without a mobile phone are 3.35 times more likely not to receive OPV1. Children of mothers who do not read newspapers are 2.75 times more likely to delay OPV3 immunization, compared to mothers who read newspapers. Mothers who disagree with the positive attributes of child immunization and do not perceive ‘child immunization is essential these days’ are much more (25 times more) likely to delay or not take their children for OPV immunization on time; mothers who perceived other benefits (non-health) of immunization are three times more likely to timely immunize their children.

## Discussion

### Limitations

Data collection and analysis have several limitations. First, the data was limited to the work areas of CGPP India in selected high-risk polio areas (villages and urban wards) of study districts where specific social mobilization interventions have been executed to improve polio immunization coverage. The social mobilization interventions of CGPP might have influenced the knowledge, attitude, and perceptions of the community towards immunization. Second, the data excluded children without any record of immunization in the form of an RI card. At the time of stratification, children who received three doses of OPV after the age of 4 months were excluded as they did not fit under the original study criteria of either Doers or Non-doers. The survey followed a case-control type design, excluded specific group children, and precluded calculation of attributable risk for identified risk factors. Third, some factors like media habits of respondents, exposure to immunization site or IEC materials did not include detailed information about investigated habits or quality of exposure; media habits of respondents were investigated as the current practice of listening/watching/reading specific media. Hence, the results based on this data are to be generalized with caution, and it should be remembered that the results are solely based on the demand side factors collected only from caregivers.

### Determinants of timely immunization of OPV3

This study found that time-appropriate vaccination of OPV3 is determined by following eight demand-side factors: a) Father’s education, b) Mother’s income generation status, c) Mother’s visit to immunization site, d) Mother’s newspaper reading habits, e) Mother’s perception about ‘positive attributes/essentiality of immunization’, f) Mother’s perception about non-health benefits of immunization, g) Household wealth status, and h) Households having a mobile phone. Out of these eight determinants, ‘Mother’s income generation status’ is a most reliable predictor with a smaller degree of variability. However, father’s education is the strongest predictor of timely OPV3 vaccination (OR = 39), but it is likely to have a wide range of variation (95% CI is 7, 216). Similarly, other six determinants also have more width of variation. This observed degree of a greater variability inherent in most the estimates might be a result of small size (68) among a group of samples (stratum 2- Children without OPV1 vaccination).

Gender of children and religion are the known factors affecting child immunization in India, but this study did not find an association of these factors with timely OPV3 immunization. No association was observed between gender and timely OPV3 vaccination; this could be due to the changing social norms and the interventions of CGPP’s SM Net activities in the study area. Recent NFHS-4 data from Uttar Pradesh [50] showed a marginal variation of two percentage points between the OPV3 immunization of boys and girls, whereas, OPV3 coverage varied by five percentage points between the children of Hindu (69%) and Muslim religion (64%) [50]. The majority Muslim population could explain not finding an association between religion and OPV3 immunization.

This study found that influence of maternal education on OPV3 immunization is of low significance in the presence of father’s education and other household-level characteristics like wealth status and ownership of a mobile phone. However, determining factors in timely immunization were mothers’ empowerment-related factors like income generation status, newspaper reading habit, and going out for child immunization. Mothers’ perceptions towards child immunization, particularly believing in ‘positive attributes/essentiality of immunization’ and ‘non-health benefits like ‘immunization, ensured general well-being of a child’ also determines timely vaccination of children.

The background characteristics of parents and the profile of households are key determinants of timely OPV immunization, the study found. Other determinants are more sensitive to change through the specific program efforts. Immunization programs can tailor their communication strategies and create a sense of urgency to address the issue of timeliness in child immunization. This issue needs to be conveyed to service providers, including vaccinators and shared with the community.

Although more than half of the mothers from all three strata of this study do not agree with a statement *‘There is no particular age, week or day, we can get the child immunized at any time’*; program communication efforts must continue to inform the community about immunization with a greater focus on timeliness.

Children from households with lower wealth quintile or less educated parents should be the focus of personalized messaging and the communication tools and techniques should be appropriate to illiterate or less educated populations. Study findings signify the necessity of involving males, as the children of educated fathers are more likely to be immunized on time. The immunization program must attempt to reach the male members of the community and convey the significance of immunizing children on time.

Since two-thirds (67%) of households have a mobile phone and it is one of the predictors of timely OVP3 immunization, the immunization program can attempt to reach these households by sending personalized messages or reminders about the immunization. Even though only less than half (44%) of the respondent mothers can use the phone on their own and majority of them (92%) could not use the phone for sending text messages, it is observed that at least one person in most families checks for messages or missed calls daily. With the growing reach of the internet and reduced-price availability of internet services, social media usage is increasing rapidly in India [51, 52]. Hence, audio-visual messaging would be helpful to mothers and family members in understanding the importance of timely immunization.

The key message of communication should be centered on the significance of timeliness in child immunization and its benefits. Alongside the core benefits of immunization, communication materials must include the messages related to other benefits and positive attributes of immunization like ‘Immunization prevents children from a physical deformity,’ ‘Immunization ensures the general well-being of children,’ and ‘Child immunization is essential these days.’ Mothers’ exposure to immunization activities is another predictor of timeliness, and the immunization program should motivate caregivers for visiting the immunization site, along with making the community aware of the place (venue), day and timing of immunization services. Frontline health workers, including CMCs, should prioritize age-appropriate vaccination. Finally, to overcome the stated limitations of the study, one may conduct in-depth investigations and assess the role of service delivery and service providers on timeliness. Further research would be useful to estimate attributable risk for various risk factors of ‘No vaccination’ and ‘delayed vaccination’ as well as assessing frontline vaccinators’ knowledge, perceptions, and practices about timely immunization.

## Conclusions

The study identified eight demand-side predictors of timely OPV3 immunization, and some of them are somewhat similar to the conventionally known predictors of routine childhood immunization, such as the socio-economic characteristics of caregivers. Key determinants are background characteristics of parents, like education and income generation status of mothers as well as the wealth status of households. Other important determinants are the caregivers’ perceptions towards the benefits or positive attributes of immunization. The immunization program needs to consider these factors when shaping communication strategies. There is also the need to create a sense of urgency among service providers and clients to address the issue of timeliness in child immunization. The immunization program must attempt to reach male members of the community and convey the significance of immunizing children on time. Every component of program communication must emphasize the need for timeliness. Along with routine monitoring of RI coverage, immunization programs should also track timeliness in the performance of child immunization. Further investigation would be useful to assess the attributable risks of various risk factors of under-immunization and vaccinators understanding about timely immunization.

## Additional files

Additional file 1: Appendix Tables. **Table S1.** Sample size covered in the doers and non-doers survey by study district. **Table S2.** Socio-demographic characteristics of index children, respondent mothers, husbands of respondents and surveyed households by three independent samples of the survey. **Table S3.** Ownership/access to household level facilities/services by three independent samples of doers and non-doers survey. **Table S4.** Wealth index of surveyed households by three independent samples of the survey. **Table S5.** Media habits of respondents by three independent samples of the survey. **Table S6.** Lifestyle related practices of respondents by three independent samples of the survey. **Table S7.** Exposure of respondents to immunization sites and interactions with frontline workers by three independent samples of the survey. **Table S8.** Information sources of respondents on getting their children immunized by three independent samples of the survey. **Table S9.** Exposure of respondents to selected IEC materials of CGPP India by three independent samples of the survey. **Table S10.** Awareness level of respondents about vaccine-preventable diseases by three independent samples of the survey. **Table S11.** Distribution of non-doers respondents by stated reasons behind not vaccinating children on time. **Table S12.** Components that determined from 12 items on the importance of child immunization, derived through principal. Component. analysis. **Table S13.** Details of five components determined from 27 items on attributes of the place of delivery, living environment, and child immunization, through principal component analysis. (DOC 375 kb)

Additional file 2: Additional text - Definitions and descriptions. (DOCX 20 kb)

## Abbreviations

CGPP: CORE Group Polio Project
CMC: Community mobilization coordinator
DPT: Diphtheria, Pertussis and Tetanus
FIC: Full immunization coverage
IEC: Information, Education and Communication
IPV: Inactivated polio vaccine
MCV: Measles-containing vaccine
OPV: Oral polio vaccine (given under routine immunization program)
OPV1: First dose of oral polio vaccine (given under routine immunization program)
OPV2: Second dose of oral polio vaccine (given under routine immunization program)
OPV3: Third dose of oral polio vaccine (given under routine immunization program)
PCA: Principal Component Analysis
RI: Routine immunization
VPD: Vaccine-preventable disease

## Footnotes

1 The CORE Group is a USAID supported umbrella organization of nongovernmental organizations (NGOs) that collaborate on international health and development programs. In India, the CORE Group Polio Project (CGPP) works across twelve districts in the state of Uttar Pradesh (UP) and is a member of the Social Mobilization Network (SM Net) in India along with UNICEF, Rotary, the Indian Government’s and WHO/National Polio Surveillance Project (NPSP) as partners. The SM Net supports polio eradication through the following efforts: identifying high-risk areas and working with underserved communities in planning, implementing and monitoring social mobilization and other immunization activities. The primary effort of the SM Net is carried out by a three-level network of mobilizers (community, block and district level).

2 RI Monitoring is a concurrent evaluation of RI program. In Uttar Pradesh, India it is jointly performed by the Government of U.P, WHO, UNICEF, CORE (CGPP India) and other stakeholders.

3 Doers and Non-doers survey – is a quantitative tool of barrier analysis, widely recommended for designing behavior change strategies. This tool helps in identifying behavioral determinants (barriers and facilitating factors) of a particular behavior [44].

4 The Community Mobilization Coordinators (CMCs) are deployed in high-risk areas (HRAs) for polio. As the backbone of the SM Net, they are assigned responsibility for mobilizing about 500 households in either a rural or an urban area, and keep records of the immunization status of all children less than five years of age in those households. CMC areas are groups of communities in a block where the SM Net is deployed. See Weiss et al. (2013) for more details about the CMCs and CMC areas [14].

5 Full immunization coverage (FIC) measured as ‘Proportion of children aged 12-23 months vaccinated with BCG, 3 doses of OPV, 3 doses of DPT and first dose of MCV’.

6 RI cards – Immunization card also known as RI card or MCP card (Mother and Child Protection Card). In India, children receive RI cards from the public (government) and private health delivery system. In government health system, the pubic health care provider (e.g. Auxiliary Nurse Midwife) provides MCP cards to pregnant women at the time of antenatal registration. The MCP card has a provision for recording the vaccination status and maternal health care services (including tetanus toxoid injection); whereas most of the RI cards provided by private institutions contain only immunization status. Whenever a child is vaccinated, the vaccinator records the date of immunization in the card.

7 Index children – were the 6-11 months old children who received OPV3 before the age of 4 months or were yet to receive the third dose (received only first or second dose of OPV). Children who received OPV3 after the age of 4 months were excluded from the study.

8 Information Education and Communication (IEC) materials are the tools used by communicators to support the target audience in decision making about behaviors. CGPP India provides various types of print and electronic (audio-visual) materials to CMCs.
