# Supplementary material for: Demand-side determinants of timely vaccination of oral polio vaccine in social mobilization network areas of CORE Group polio project in Uttar Pradesh, India
Source: BMC Infect Dis. 2018 May 16;18:222. doi: 10.1186/s12879-018-3129-2 (PMC5956729; doi:10.1186/s12879-018-3129-2)
Supplement: Supplementary file 1 — Appendix Tables. Table S1. Sample size covered in the doers and non-doers survey by study district. Table S2. Socio-demographic characteristics of index children, respondent mothers, husbands of respondents and surveyed households by three independent samples of the survey. Table S3. Ownership/access to household level facilities/services by three independent samples of doers and non-doers survey. Table S4. Wealth index of surveyed households by three independent samples of the survey. Table S5. Media habits of respondents by three independent samples of the survey. Table S6. Lifestyle related practices of respondents by three independent samples of the survey. Table S7. Exposure of respondents to immunization sites and interactions with frontline workers by three independent samples of the survey. Table S8. Information sources of respondents on getting their children immunized by three independent samples of the survey. Table S9. Exposure of respondents to selected IEC materials of CGPP India by three independent samples of the survey. Table S10. Awareness level of respondents about vaccine-preventable diseases by three independent samples of the survey. Table S11. Distribution of non-doers respondents by stated reasons behind not vaccinating children on time. Table S12. Components that determined from 12 items on the importance of child immunization, derived through principal. Component. analysis. Table S13. Details of five components determined from 27 items on attributes of the place of delivery, living environment, and child immunization, through principal component analysis. (DOC 375 kb) [file 12879_2018_3129_MOESM1_ESM.doc]

**Appendix Tables**

**Appendix Table 1: Sample size covered in the doers and non-doers survey by study district**

| **Study district** | **Interviews of mothers of children aged 6-11 months who received:** | | | |
| --- | --- | --- | --- | --- |
|  | No OPV1 immunization  **(Non-doers- A)**  No. (%) | OPV1/OPV2 immunization  **(Non-doers – B)**  No. (%) | Timely immunization of OPV3 **(Doers)**  No. (%) | Total  **(Doers & Non-doers)**  No. (%) |
| Rampur | 9 (13.2) | 70 (26.8) | 66 (26.0) | 145 (24.9) |
| Saharanpur | 10 (14.7) | 25 (9.6) | 129 (50.8) | 164 (28.1) |
| Sambhal | 49 (72.1) | 166 (63.6) | 59 (23.2) | 274 (47.0) |
| Total (3 study districts) | 68 (100.0) | 261 (100.0) | 254 (100.0) | 583 (100.0) |

**Appendix Table 2 - Socio-demographic characteristics of index children, respondent mothers, husbands of respondents and surveyed households by three independent samples of the survey**

| **Characteristics** |  | **Percentage^♦^ of mothers of children with:** | | | **Overall**  ***(n=583)*** | **p value^$^** |
| --- | --- | --- | --- | --- | --- | --- |
|  |  | **No OPV1** immunization  *(n=68)* | **OPV1/OPV2** immunization  *(n=261)* | Timely **OPV3** immunization  *(n=254)* |  |  |
| ***Characteristics of index children*** | | | | |  |  |
| Gender^[a]^ | Boy | 47.9 | 54.5 | 54.5 | 53.5 | 0.345 |
|  | Girl | 52.1 | 45.5 | 45.5 | 46.5 |  |
| Place of birth | Non-institution (Home) | 46.9 | 42.4 | 44.1 | 43.6 | 0.402 |
|  | Institution – Govt. hospital | 29.7 | 26.9 | 23.3 | 26.0 |  |
|  | Institution - Private hospital | 23.3 | 30.7 | 32.6 | 30.4 |  |
| ***Characteristics of mothers (respondents) of index children*** | | | | |  |  |
| Age (in years)^[b]^ | <20 years | 5.9 | 1.9 | 1.3 | 2.2 | 0.069 |
|  | 20-24 years | 29.0 | 34.4 | 38.8 | 35.3 |  |
|  | 25-29 years | 31.0 | 37.7 | 36.1 | 36.2 |  |
|  | 30-34 years | 28.9 | 17.8 | 16.2 | 18.8 |  |
|  | >34 years | 5.2 | 8.2 | 7.6 | 7.5 |  |
| Education level | No formal education | 70.6 | 69.1 | 50.2 | 62.4 | 0.013* |
|  | Grade (Standard) 1- 4 | 6.9 | 3.9 | 3.8 | 4.3 |  |
|  | Grade (Standard) 5-9 | 13.9 | 16.3 | 22.4 | 18.2 |  |
|  | Grade (Standard) 10 and above | 8.6 | 10.7 | 23.6 | 15.1 |  |
| Place of income generation | Income generation work from home | 1.7 | 3.1 | 8.6 | 4.9 | <0.001*** |
|  | Income generation work from outside home | 1.7 | 1.4 | 1.6 | 1.5 |  |
|  | No income generation work (stay at home) | 96.6 | 95.5 | 89.8 | 93.6 |  |
| Religion | Muslim | 91.4 | 83.3 | 80.1 | 83.2 | 0.291 |
|  | Non-Muslim (Hindu & others) | 8.6 | 16.7 | 19.9 | 16.8 |  |
| ***Characteristics of fathers of index children*** | | | | |  |  |
| Age (in years) ^[c]^ | <25 years | 6.8 | 9.5 | 10.5 | 9.5 | 0.389 |
|  | 25-29 years | 38.4 | 36.6 | 38.0 | 37.4 |  |
|  | 30-34 years | 25.0 | 30.2 | 32.4 | 30.3 |  |
|  | >34 years | 29.8 | 23.7 | 19.1 | 22.8 |  |
| Education level | No formal education | 68.0 | 51.7 | 35.5 | 48.0 | 0.005** |
|  | Grade (Standard) 1- 4 | 5.2 | 4.9 | 8.1 | 6.1 |  |
|  | Grade (Standard) 5-9 | 24.2 | 22.8 | 27.9 | 24.9 |  |
|  | Grade (Standard) 10 and above | 2.6 | 20.6 | 28.5 | 21.0 |  |
| Place of income generation | Income generation work from home | 1.8 | 6.3 | 13.4 | 8.2 | 0.148 |
|  | Income generation work from outside home | 98.2 | 92.8 | 86.0 | 91.1 |  |
|  | No income generation work (stay at home) | 0.0 | 0.9 | 0.6 | 0.7 |  |
| ***Characteristics of surveyed households*** | | | | |  |  |
| Family type | Nuclear | 52.1 | 52.6 | 46.9 | 50.5 | 0.479 |
|  | Joint | 47.9 | 47.4 | 53.1 | 49.5 |  |
| Respondents staying with mothers-in-law | No | 59.7 | 54.5 | 49.8 | 53.5 | 0.394 |
|  | Yes | 40.3 | 45.5 | 50.2 | 46.5 |  |
| Housing type | Semi-*pucca* or *kuchha* | 42.5 | 42.5 | 24.0 | 35.7 | 0.050* |
|  | *Pucca* | 57.5 | 57.5 | 76.0 | 64.3 |  |

**^♦^** Percentages are weighted by population size, adjusted for stratification, and clustering

Missing data: [a] = 7 (1.2%); [b] = 1 (0.2%); [c] = 1 (0.2%)

^$^ p value based on chi-square test; *statistically significant at p < 0.05; ** p < 0.01; ***p < 0.001

**Appendix Table 3 - Ownership/access to household level facilities/services by three independent samples of doers and non-doers survey**

| **Household level facilities/services** |  | **Percentage^♦^ of mothers of children with:** | | | **Overall**  ***(n=583)*** | **p value^$^** |
| --- | --- | --- | --- | --- | --- | --- |
|  |  | **No OPV1** immunization  *(n=68)* | **OPV1/OPV2** immunization  *(n=261)* | Timely **OPV3** immunization  *(n=254)* |  |  |
| Air conditioner | Do not have | 97.4 | 98.3 | 94.1 | 96.7 | 0.082 |
|  | Have | 2.6 | 1.7 | 5.9 | 3.3 |  |
| Car | Do not have | 100.0 | 98.6 | 98.3 | 98.7 | 0.592 |
|  | Have | 0.0 | 1.4 | 1.7 | 1.3 |  |
| Washing Machine | Do not have | 88.8 | 83.2 | 72.0 | 79.9 | 0.047* |
|  | Have | 11.2 | 16.8 | 28.0 | 20.1 |  |
| Refrigerator | Do not have | 83.6 | 77.4 | 69.7 | 75.4 | 0.101 |
|  | Have | 16.4 | 22.6 | 30.3 | 24.6 |  |
| Color TV | Do not have | 62.4 | 45.9 | 38.1 | 45.3 | 0.053 |
|  | Have | 37.6 | 54.1 | 61.9 | 54.7 |  |
| Two Wheeler | Do not have | 66.8 | 62.2 | 47.1 | 57.3 | 0.018* |
|  | Have | 33.2 | 37.8 | 52.9 | 42.7 |  |
| Pressure Cooker | Do not have | 16.9 | 13.8 | 14.4 | 14.4 | 0.790 |
|  | Have | 83.1 | 86.2 | 85.6 | 85.6 |  |
| Toilet in own home/Compound | Do not have | 5.8 | 9.6 | 9.4 | 9.0 | 0.441 |
|  | Have | 94.2 | 90.4 | 90.6 | 91.0 |  |
| Dining Table | Do not have | 75.8 | 75.7 | 76.4 | 76.0 | 0974 |
|  | Have | 24.2 | 24.3 | 23.6 | 24.0 |  |
| LPG stove | Do not have | 44.8 | 44.2 | 34.7 | 40.8 | 0.199 |
|  | Have | 55.2 | 55.8 | 65.3 | 59.2 |  |
| Ceiling Fan | Do not have | 9.3 | 6.5 | 6.3 | 6.8 | 0.497 |
|  | Have | 90.7 | 93.5 | 93.7 | 93.2 |  |
| Electricity connection | Do not have | 5.8 | 8.6 | 6.3 | 7.4 | 0.418 |
|  | Have | 94.2 | 91.4 | 93.7 | 92.6 |  |

**^♦^** Percentages are weighted by population size, adjusted for stratification, and clustering

^$^ p value based on chi-square test; *statistically significant at p < 0.05; ** p < 0.01; ***p < 0.001

**Appendix Table 4 – Wealth index of surveyed households by three independent samples of the survey**

| **Wealth quintile** | **Percentage^♦^ of mothers of children with:** | | | **Overall**  ***(n=583)*** | **p value^$^** |
| --- | --- | --- | --- | --- | --- |
|  | **No OPV1** immunization  *(n=68)* | **OPV1/OPV2** immunization  *(n=261)* | Timely **OPV3** immunization  *(n=254)* |  |  |
| Lowest | 22.0 | 26.6 | 15.0 | 21.7 | 0.006** |
| Second | 23.7 | 21.4 | 14.2 | 19.1 |  |
| Middle | 22.4 | 16.8 | 24.1 | 20.2 |  |
| Fourth | 22.4 | 19.5 | 16.6 | 18.9 |  |
| Highest | 9.5 | 15.7 | 30.1 | 20.1 |  |

**^♦^** Percentages are weighted by population size, adjusted for stratification, and clustering

^$^ p value based on chi-square test; *statistically significant at p < 0.05; ** p < 0.01; ***p < 0.001

**Appendix Table 5 – Media habits of respondents by three independent samples of the survey**

| **Media habit** |  | **Percentage^♦^ of mothers of children with:** | | | **Overall**  ***(n=583)*** | **p value^$^** |
| --- | --- | --- | --- | --- | --- | --- |
|  |  | **No OPV1** immunization  *(n=68)* | **OPV1/OPV2** immunization  *(n=261)* | Timely **OPV3** immunization  *(n=254)* |  |  |
| Television | Do not watch | 41.7 | 35.4 | 31.8 | 35.0 | 0.429 |
|  | Watch | 58.3 | 64.6 | 68.2 | 65.0 |  |
| Newspaper | Do not read | 84.4 | 92.5 | 77.5 | 85.9 | 0.013* |
|  | Read | 15.6 | 7.5 | 22.5 | 14.1 |  |
| Radio | Do not listen | 93.3 | 90.1 | 93.5 | 91.8 | 0.471 |
|  | Listen | 6.7 | 9.9 | 6.5 | 8.2 |  |
| Cinema | Do not watch (Don’t go to cinema hall) | 100.0 | 99.8 | 100.0 | 99.9 | 0.877 |
|  | Watch (Goes to cinema Hall) | 0.0 | 0.2 | 0.0 | 0.1 |  |
| Anyone media | No exposure to any of the above media | 40.9 | 33.2 | 28.6 | 32.6 | 0.212 |
|  | Exposed to at least one of the above four media(TV/Newspaper/Radio/Cinema) | 59.1 | 66.8 | 71.4 | 67.4 |  |

**^♦^** Percentages are weighted by population size, adjusted for stratification, and clustering

^$^ p value based on chi-square test; *statistically significant at p < 0.05; ** p < 0.01; ***p < 0.001

**Appendix Table 6 – Lifestyle related practices of respondents by three independent samples of the survey**

| **Lifestyle-related practices** |  | **Percentage^♦^ of mothers of children with:** | | | **Overall**  ***(n=583)*** | **p value^$^** |
| --- | --- | --- | --- | --- | --- | --- |
|  |  | **No OPV1** immunization  *(n=68)* | **OPV1/OPV2** immunization  *(n=261)* | Timely **OPV3** immunization  *(n=254)* |  |  |
| Respondents visited a nearby town (e.g. district town) in the last one month | No | 76.4 | 85.6 | 80.4 | 82.4 | 0.156 |
|  | Yes | 23.6 | 14.4 | 19.6 | 17.6 |  |
| Respondents visited a big metro town (like Delhi) in the last 2 – 3 years | No | 93.9 | 98.3 | 93.5 | 95.9 | 0.060 |
|  | Yes | 6.1 | 1.7 | 6.5 | 4.1 |  |
| Respondents’ having a lot of friends and she is a leader (*Mukhia*) of friends | No | 100.0 | 98.8 | 99.0 | 99.1 | 0.692 |
|  | Yes | 0.0 | 1.2 | 1.0 | 0.9 |  |
| Respondents having a family member, who works in a town (outside of village) | No | 94.0 | 91.6 | 87.4 | 90.4 | 0.114 |
|  | Yes | 6.0 | 8.4 | 12.6 | 9.6 |  |
| Respondents having a family member, who works in a big metro town (like Delhi) | No | 89.8 | 96.1 | 95.0 | 94.8 | 0.029* |
|  | Yes | 10.2 | 3.9 | 5.0 | 5.2 |  |
| A family member who works in a town/big metro town visit to respondents regularly (i.e., at least once in 2 years) | No | 88.1 | 91.9 | 87.5 | 89.8 | 0.092 |
|  | Yes | 11.9 | 8.1 | 12.5 | 10.2 |  |
| Respondents having a mobile phone in the household | No | 36.8 | 37.8 | 23.6 | 32.5 | 0.002** |
|  | Yes | 63.2 | 62.2 | 76.4 | 67.5 |  |
| Respondents know how to use a mobile phone, use it independently | No | 65.6 | 61.9 | 43.3 | 55.6 | 0.008** |
|  | Yes | 34.4 | 38.1 | 56.7 | 44.4 |  |
| Respondents know how to use mobile for sending messages (SMS) | No | 94.8 | 96.5 | 84.8 | 92.0 | 0.002** |
|  | Yes | 5.2 | 3.5 | 15.2 | 8.0 |  |

**^♦^** Percentages are weighted by population size, adjusted for stratification, and clustering

^$^ p value based on chi-square test; *statistically significant at p < 0.05; ** p < 0.01; ***p < 0.001

**Appendix Table 7 – Exposure of respondents to immunization sites and interactions with frontline workers by three independent samples of the survey**

| **Variable** |  | **Percentage^♦^ of mothers of children with:** | | | **Overall**  ***(n=583)*** | **p value^$^** |
| --- | --- | --- | --- | --- | --- | --- |
|  |  | **No OPV1** immunization  *(n=68)* | **OPV1/OPV2** immunization  *(n=261)* | Timely **OPV3** immunization  *(n=254)* |  |  |
| Visit to a routine immunization (RI) site | Never visited | 56.5 | 20.0 | 12.8 | 22.4 | <0.001*** |
|  | Ever visited | 43.5 | 80.0 | 87.2 | 77.6 |  |
| Visit to a polio (SIA) booth | Never visited | 60.1 | 21.6 | 18.5 | 25.7 | <0.001*** |
|  | Ever visited | 39.9 | 78.4 | 81.5 | 74.3 |  |
| Visit to an immunization site (RI/SIA) | Never visited to RI/SIA site | 55.7 | 19.5 | 12.8 | 22.1 | <0.001*** |
|  | Ever visited to RI/SIA site | 44.3 | 80.5 | 87.2 | 77.9 |  |
| Discussion with local health care providers on the importance of child immunization | Never discussed | 14.6 | 5.9 | 3.2 | 6.1 | <0.001*** |
|  | Ever discussed | 85.4 | 94.1 | 96.8 | 93.9 |  |
| Discussion with CMCs on importance of child immunization | Never discussed | 14.6 | 9.3 | 9.8 | 10.2 | 0.426 |
|  | Ever discussed | 85.4 | 90.7 | 90.2 | 89.8 |  |

**^♦^** Percentages are weighted by population size, adjusted for stratification, and clustering

^$^ p value based on chi-square test; *statistically significant at p < 0.05; ** p < 0.01; ***p < 0.001

**Appendix Table 8 – Information sources of respondents on getting their children immunized by three independent samples of the survey**

| **Source of information on child immunization** |  | **Percentage^♦^ of mothers of children with:** | | | **Overall**  ***(n=583)*** | **p value^$^** |
| --- | --- | --- | --- | --- | --- | --- |
|  |  | **No OPV1** immunization  *(n=68)* | **OPV1/OPV2** immunization  *(n=261)* | Timely **OPV3** immunization  *(n=254)* |  |  |
| Herself (Respondent mother) | No | 85.5 | 82.1 | 90.1 | 85.5 | 0.222 |
|  | Yes | 14.5 | 17.9 | 9.9 | 14.5 |  |
| Advertisements in newspaper /TV/radio/ group SMS | No | 98.3 | 98.8 | 93.4 | 96.8 | 0.057 |
|  | Yes | 1.7 | 1.2 | 6.6 | 3.2 |  |
| Family members/relatives/ friends/ neighbors | No | 97.4 | 94.7 | 87.5 | 92.4 | 0.152 |
|  | Yes | 2.6 | 5.3 | 12.5 | 7.6 |  |
| Community Mobilization Coordinators (CMC) | No | 18.1 | 14.3 | 8.4 | 12.7 | 0.067 |
|  | Yes | 81.9 | 85.7 | 91.6 | 87.3 |  |
| Aanganwadi worker/ health worker/ ASHA/ ANM/ Doctor | No | 98.5 | 97.3 | 81.2 | 91.6 | 0.065 |
|  | Yes | 1.5 | 2.7 | 18.8 | 8.4 |  |

**^♦^** Percentages are weighted by population size, adjusted for stratification, and clustering

^$^ p value based on chi-square test; *statistically significant at p < 0.05; ** p < 0.01; ***p < 0.001

**Appendix Table 9 – Exposure of respondents to selected IEC materials of CGPP India by three independent samples of the survey**

| IEC materials of CGPP India |  | **Percentage^♦^ of mothers of children with:** | | | **Overall**  ***(n=583)*** | **p value^$^** |
| --- | --- | --- | --- | --- | --- | --- |
|  |  | **No OPV1** immunization  *(n=68)* | **OPV1/OPV2** immunization  *(n=261)* | Timely **OPV3** immunization  *(n=254)* |  |  |
| CMC Potli | Not exposed | 78.6 | 73.6 | 56.9 | 68.2 | 0.020* |
|  | Exposed | 21.4 | 26.4 | 43.1 | 31.8 |  |
| Leaflet | Not exposed | 99.2 | 89.4 | 87.5 | 90.1 | 0.013* |
|  | Exposed | 0.8 | 10.6 | 12.5 | 9.9 |  |
| Flash Card | Not exposed | 95.8 | 90.9 | 79.1 | 87.2 | 0.002** |
|  | Exposed | 4.2 | 9.1 | 20.9 | 12.8 |  |
| Flip Book (*Aao Jane*) | Not exposed | 88.8 | 82.8 | 69.5 | 78.8 | 0.003** |
|  | Exposed | 11.2 | 17.2 | 30.5 | 21.2 |  |
| Congratulatory card (*Badhai card*) | Not exposed | 52.5 | 37.8 | 45.5 | 42.6 | 0.261 |
|  | Exposed | 47.5 | 62.2 | 54.5 | 57.4 |  |
| Exposure to at least one of above five IEC materials | Not exposed | 39.6 | 29.8 | 25.1 | 29.5 | 0.041* |
|  | Exposed | 60.4 | 70.2 | 74.9 | 70.5 |  |

**^♦^** Percentages are weighted by population size, adjusted for stratification, and clustering

^$^ p value based on chi-square test; *statistically significant at p < 0.05; ** p < 0.01; ***p < 0.001

**Appendix Table 10 – Awareness level of respondents about vaccine-preventable diseases by three independent samples of the survey**

| **Vaccine-preventable disease** |  | **Percentage^♦^ of mothers of children with:** | | | **Overall**  ***(n=583)*** | **p value^$^** |
| --- | --- | --- | --- | --- | --- | --- |
|  |  | **No OPV1** immunization  *(n=68)* | **OPV1/OPV2** immunization  *(n=261)* | Timely **OPV3** immunization  *(n=254)* |  |  |
| Diphtheria | Not heard | 57.3 | 50.8 | 47.8 | 50.6 | 0.589 |
|  | Heard | 42.7 | 49.2 | 52.2 | 49.4 |  |
| Tuberculosis | Not heard | 4.0 | 1.9 | 1.6 | 2.1 | 0.560 |
|  | Heard | 96.0 | 98.1 | 98.4 | 97.9 |  |
| Polio | Not heard | 6.0 | 3.9 | 5.8 | 4.9 | 0.467 |
|  | Heard | 94.0 | 96.1 | 94.2 | 95.1 |  |
| Tetanus | Not heard | 5.8 | 4.1 | 5.3 | 4.8 | 0.811 |
|  | Heard | 94.2 | 95.9 | 94.7 | 95.2 |  |
| Pertussis | Not heard | 16.5 | 7.9 | 14.0 | 11.3 | 0.326 |
|  | Heard | 83.5 | 92.1 | 86.0 | 88.7 |  |
| Jaundice | Not heard | 6.6 | 1.9 | 2.9 | 2.9 | 0.096 |
|  | Heard | 93.4 | 98.1 | 97.1 | 97.1 |  |
| Diarrhea | Not heard | 5.2 | 1.4 | 1.6 | 2.0 | 0.146 |
|  | Heard | 94.8 | 98.6 | 98.4 | 98.0 |  |
| Measles | Not heard | 7.8 | 2.4 | 11.2 | 6.4 | 0.250 |
|  | Heard | 92.2 | 97.6 | 88.8 | 93.6 |  |

**^♦^** Percentages are weighted by population size, adjusted for stratification, and clustering

^$^ p value based on chi-square test; *statistically significant at p < 0.05; ** p < 0.01; ***p < 0.001

**Appendix Table 11 – Distribution of non-doers respondents by stated reasons behind not vaccinating children on time**

| **Reasons behind not vaccinating children** | | **Percentage^♦^ of mothers of children with:** | | | **p value^$^** |
| --- | --- | --- | --- | --- | --- |
|  |  | **No OPV1** immunization  *(n=68)* | **OPV1/OPV2** immunization  *(n=261)* | **All Non-doers**  ***(n=329)*** |  |
| Child was sick | Not stated | 56.1 | 57.9 | 57.5 | 0.761 |
|  | Stated | 43.9 | 42.1 | 42.5 |  |
| Child was out of home/village | Not stated | 76.9 | 79.4 | 78.9 | 0.433 |
|  | Stated | 23.1 | 20.6 | 21.1 |  |
| Unaware/carelessness of parents (Parents had/have no time for child immunization; Not aware of the schedule of child immunization; Nobody reminded about the due vaccine; Opposed by family members) | Not stated | 88.9 | 92.4 | 91.7 | 0.258 |
|  | Stated | 11.1 | 7.6 | 8.3 |  |
| Perceived negative effects (Immunization causes discomfort to the child/Make child irritate/Gives pain to child/ Child cries; Immunization does not prevent diseases/ Do not trust the vaccine - had measles; Injections are septic) | Not stated | 83.2 | 86.1 | 85.5 | 0.474 |
|  | Stated | 16.8 | 13.9 | 14.5 |  |
| Bad experience - Child feel ill/became weak post vaccination | Not stated | 100.0 | 97.4 | 98.0 | 350 |
|  | Stated | 0.0 | 2.6 | 2.0 |  |
| Operational issues -Vaccinator was absent/RI Session did not happen | Not stated | 98.3 | 98.8 | 98.7 | 0.493 |
|  | Stated | 1.7 | 1.2 | 1.3 |  |
| Stated at least one reason | Not stated | 12.0 | 16.1 | 15.2 | 0.388 |
|  | Stated | 88.0 | 83.9 | 84.8 |  |

**^♦^** Percentages are weighted by population size, adjusted for stratification, and clustering

^$^ p value based on chi-square test; *statistically significant at p < 0.05; ** p < 0.01; ***p < 0.001

**Appendix Table 12 – Components that determined from 12 items on the importance of child immunization, derived through principal component analysis**

| **Statements on the importance of child immunization** | **Item/factor loading**[[1]](#footnote-2) | **Labeling of components derived through PCA** | **Cronbach’s alfa** | **Mean score (SD)** |
| --- | --- | --- | --- | --- |
| I.5.To build strength and stamina of the child | 0.779 | **Component 1 -** Perceived core benefits of immunization | 0.81 | 65.29  (23.57) |
| I.7.To prevent the child from fatal (*Jaanleva*) diseases | 0.773 |
| I.3.To prevent the child from common ailments like cold, cough, fever, etc. | 0.772 |
| I.6.To build resistance against diseases | 0.757 |
| I.4.To prevent the child from all diseases | 0.659 |
| I.9.Do not know whether vaccinations work, but no harm in trying them | -0.899 | **Component 2 –** Perceived no harm in immunization and have trust in govt. initiatives | 0.74 | 29.96  (31.36) |
| I.8.Government invests so much money & effort; there must be some benefit although I do not know the exact benefit | -0.853 |
| I.2.To prevent the child from physical deformity | 0.875 | **Component 3** – Perceived other (non-health) benefits of immunization | 0.68 | 72.74  (22.30) |
| I.1.To ensure general well-being of the child | 0.847 |
| I.10.Educated mothers/families desire to vaccinate their child | 0.905 | **Component 4** –Perceived that educated/ aware parents go for immunization | 0.75 | 44.27  (22.87) |
| I.11.General awareness-as their families are exposed to outside world | 0.809 |
| I.12.Well informed mothers desire to vaccinate their child | 0.598 |
| Model explains 67.7% of variance (number of components were determined through Eigenvalues>1); n = 583 | | | | |

**Appendix Table 13 – Details of five components determined from 27 items on attributes of the place of delivery, living environment, and child immunization, through principal component analysis**

| **Statements related to attributes of the place of delivery, living environment, and child immunization** | **Item/factor loading** | **Labeling of components derived through PCA** | **Cronbach’s alfa** | **Mean score (SD)** |
| --- | --- | --- | --- | --- |
| I.9 Immunization has more advantages than disadvantages | 0.784 | **Component 1 –** Positive attributes/ benefits of child immunization and it is essentials these days (in polluted environment/ contaminated eatables & water) | 0.80 | 3.20  (0.88) |
| I.8 Immunization has a lot of benefits for a child | 0.770 |
| I.7 It is very important to have your child immunized these days | 0.689 |
| I.5 These days, there is a lot of pollution in the environment | 0.628 |
| I.6 All eatables and water are contaminated are not as healthy to be consumed as compared to earlier times | 0.601 |
| I.11 Immunization stops 100% fatal diseases that they are meant for | 0.574 |
| I.14 Both parents (mother & father) should ensure that they get their child immunized | 0.558 |
| I.24 Polio, two drops are okay, but there is no need for immunization | 0.762 | **Component 2** – Negative attributes/ no benefit of child immunization | 0.72 | 2.02  (0.59) |
| I.23 There is no need for immunization if a child is kept clean / hygiene is ensured | 0.666 |
| I.22 There is no need for immunization if the child is given good nutritious diet | 0.629 |
| I.21 Immunization is a modern day fashion/fad | 0.576 |
| I.12 Immunization has no benefit; it is all up to the GOD to keep the child away from diseases that the immunization claim to protect from | 0.548 |
| I.10 Even if the child is immunized, he or she still get the diseases that they are meant to be protected from | 0.479 |
| I.25 There is no particular age, week or day; we can get the child immunized at any time | 0.475 |
| I.2 It is safe for the mother to deliver the child in a hospital than at home | 0.723 | **Component 3** - Positive attributes of hospital delivery and child immunization is discussed among friends | 0.71 | 3.56  (0.85) |
| I.3 It is safe for the child to be born in the hospital than at home | 0.704 |
| I.27 If an influential person in the village advocates immunization, mothers will get their child immunized for sure | 0.635 |
| I.26 My friends, whenever we meet, we discuss immunization/benefits of immunization | 0.608 |
| I.1 These days in our neighborhood more deliveries take place in hospitals than at home | 0.595 |
| I.18 Usually, families / parents who are more progressive (aware) get their child immunized | 0.817 | **Component 4 -** Child immunization is meant for educated/ moving/ progressive families | 0.69 | 3.14  (0.92) |
| I.19 Usually, families/parents that are more educated get their child immunized | 0.782 |
| I.20 Usually, families/parents that are moving around / visit/work in towns get their child immunized | 0.468 |
| I.16 Only girls should be immunized | 0.739 | **Component 5 –** Gender preference/selection in immunization | 0.79 | 1.25  (0.65) |
| I.15 Only boys should be immunized | 0.696 |
| I.4. It is hygienic for both mother and child in the hospital | **Factors excluded from the PCA model** | | | |
| I.13. Immunization is the responsibility of Government; we have no role |
| I.17. I know all the benefits of Immunization |
| Model explains 54% of variance; n = 583 | | | | |

1. **Item/factor loading** in factor analysis/principal component analysis shows correlation between an item/variable and a component/factor. [↑](#footnote-ref-2)
