# Supplementary material for: Demand-side determinants of timely vaccination of oral polio vaccine in social mobilization network areas of CORE Group polio project in Uttar Pradesh, India
Source: BMC Infect Dis. 2018 May 16;18:222. doi: 10.1186/s12879-018-3129-2 (PMC5956729; doi:10.1186/s12879-018-3129-2)
Supplement: Supplementary file 2 — Additional text - Definitions and descriptions. (DOCX 20 kb) [file 12879_2018_3129_MOESM2_ESM.docx]

**Definitions and Descriptions**

**Complex samples analysis** – The complex samples analysis allows for weighting of population size (no. of households) of the study districts, CMC areas (our survey clusters) and adjusts standard errors for clustering by districts [1]. While defining the complex sample analysis plan, three independent sample groups were considered as stratum and study samples were weighted by a number of households covered by CGPP in the study districts. In the analysis plan, sample design of equal probability sampling without replacement was assumed for the estimation. Equal inclusion probabilities of samples were specified in two stages for all the three strata. Stratum wise, district-specific and overall observed levels (proportions) of OPV immunization in the monthly progress report of CGPP India were used in specifying inclusion probabilities of samples [2].

**Assessment of multicollineari**ty **among covariates** - At the first stage, pairwise (bivariate) collinearity was assessed by observing bivariate correlation coefficients. All the independent variables were observed with mild/moderate (<0.7) correlation. Further, a collinearity diagnostics test was applied to assess the multicollinearity by observing Variance Inflation Factor (VIF) statistics. All the 21 variables were observed with VIF statistics less than three; hence none of the variables were excluded from the model.

**Computation of household wealth index** - Based on 12 items related to household level ownership or access to facilities/services (presented in Appendix Table 2) and type of house, a wealth index was computed by following steps recommended for demographic and health survey[3]. After assessing the internal consistency between the 13 items, two variables, i.e., having a car and pressure cooker were excluded from the index. The final wealth index (based on 11 items) is having Cronbach’s alpha value of 0.71. Using single factor extraction in PCA analysis (explaining 28% of variance), Factor scores were generated for every household. Computed factor score was ranked and divided into five quintiles.

**Computation of composite perception score on the importance of childhood immunization** - The 12 perceptions related items (presented in Appendix Table 12) were reduced into four components using factor (principal component) analysis, with the PCA model explaining 68% variance. A composite score was developed for each of the four components using average (mean) score method, as all the 12 original statements were investigated on the same scale, i.e., 1-100 points[4]. Internal consistency of computed composite score was assessed through coefficient alfa. Computed Cronbach’s alfa of all four subscale score was above 0.67. Finally, the mean scores were converted into three categories using percentiles (quantiles) and presented in Table 1.

**Computation of composite score of respondents’ agreement on attributes of the place of delivery, living environment, and child immunization** – All 27 perception and attitudes related items were reduced to five components using factor (principal component) analysis, with a PCA model explaining 54 percent variance (see Appendix Table 13). PCA based components were further tested for internal consistency using coefficient alfa (Cronbach’s alfa). Items no. 4, 13 and 17 were removed from the model to improve the reliability of scale and also because these items were somewhat covered by the remaining 24 items. Reliability of the subscale score for component 1, 2, 3 4 and 5 was 0.80, 0.72, 0.71, 0.69 and 0.79 respectively. Further, a composite agreement score was developed for each of the five components using average (mean) score method, as all the 24 original attitudes related statements were investigated on the same scale, i.e., 1-5 points [4]. Finally, the mean scores were converted into three categories using percentiles and presented in Table 2.

**References:**

1. IBM Corporation. IBM SPSS Complex Samples 20 - Software Manual. IBM Corporation; 2011.

2. CGPP India. Monthly Progress Report - February 2014. CORE Group Polio Project; 2014 Feb.

3. Rutstein SO, Johnson K. The DHS Wealth Index [Internet]. DHS Comp. Reports No. 6. Calverton, Maryland USA, Maryland USA: ORC Macro; 2004 Aug. Report No.: DHS Comparative Reports No. 6. Available from: http://www.dhsprogram.com/pubs/pdf/CR6/CR6.pdf

4. Distefano C, Zhu M, Mîndrilă D. Understanding and using factor scores: Considerations for the applied researcher. Pract. Assessment, Res. Eval. 2009;14:1–11.
